# Understanding the contexts in which female sex workers sell sex in Kampala, Uganda: a qualitative study

**DOI:** 10.1186/s12905-024-03216-7

**Published:** 2024-06-26

**Authors:** Kenneth Roger Katumba, Mercy Haumba, Yunia Mayanja, Yvonne Wangui Machira, Mitzy Gafos, Matthew Quaife, Janet Seeley, Giulia Greco

**Affiliations:** 1 MRC/UVRI & LSHTM Uganda Research Unit, Plot 51-59 Nakiwogo Road, P.O. Box 49, Entebbe, Uganda; 2 London School of Hygiene and Tropical Medicine, London, UK; 3 IAVI Africa, Nairobi, Kenya

**Keywords:** Structural factors, Internet sex work, Social media, Risky behaviour, Uganda, HIV

## Abstract

**Background:**

Structural, interpersonal and individual level factors can present barriers for HIV prevention behaviour among people at high risk of HIV acquisition, including women who sell sex. In this paper we document the contexts in which women selling sex in Kampala meet and provide services to their clients.

**Methods:**

We collected qualitative data using semi-structured interviews. Women were eligible to participate if they were 18 years or older, self-identified as sex workers or offered sex for money and spoke Luganda or English. Ten women who met clients in venues and outdoor locations were selected randomly from a clinic for women at high risk of HIV acquisition. Ten other women who met clients online were recruited using snowball sampling. Interviews included demographic data, and themes included reasons for joining and leaving sex work, work locations, nature of relationships with clients and peers, interaction with authorities, regulations on sex work, and reported stigma. We conducted interviews over three months. Data were analysed thematically using a framework analysis approach. The coding framework was based on structural factors identified from literature, but also modified inductively with themes arising from the interviews.

**Results:**

Women met clients in physical and virtual spaces. Physical spaces included venues and outdoor locations, and virtual spaces were online platforms like social media applications and websites. Of the 20 women included, 12 used online platforms to meet clients. Generally, women from the clinic sample were less educated and predominantly unmarried, while those from the snowball sample had more education, had professional jobs, or were university students. Women from both samples reported experiences of stigma, violence from clients and authorities, and challenges accessing health care services due to the illegality of sex work. Even though all participants worked in settings where sex work was illegal and consequently endured harsh treatment, those from the snowball sample faced additional threats of cybersecurity attacks, extortion from clients, and high levels of violence from clients.

**Conclusions:**

To reduce risk of HIV acquisition among women who sell sex, researchers and implementers should consider these differences in contexts, challenges, and risks to design innovative interventions and programs that reach and include all women.

**Supplementary Information:**

The online version contains supplementary material available at 10.1186/s12905-024-03216-7.

## Introduction

### Background

Globally women who sell sex face a disproportionately large risk of HIV acquisition compared to the general population [1–3]. Among those at greatest risk are female sex workers (FSWs) in low- and middle-income countries (LMICs) who are 13.5 times more likely to acquire HIV relative to the general population [3, 4]. Research indicates that structural, interpersonal, and individual factors influence HIV prevention behaviour [4–9]. Structural factors are defined as the economic, social, political, organizational or other aspects of the environment in which women sell sex, and which might act as barriers to or facilitators of women’s HIV prevention behaviour [7, 10–14]. Interpersonal factors are those which relate to risks or protective factors between women and their clients, or intimate partners [2, 15]. Individual factors are those which relate to a woman’s individual attributes such as age of initiation into sex work, alcohol and other substance use, knowledge of HIV prevention, physical, and psychological attributes [6, 15]. Together, the structural and interpersonal factors influence the contexts in which women who sell sex work. Several structural and interpersonal factors that influence condom use among sex workers have been identified, including zoning restrictions and regulation of sex work, how women join sex work, the location where sex workers meet and provide services to clients, experiences of violent relationships with clients, and harassment by authorities and police [7]. Stigma has also been identified as an important influence on the way sex workers work and as a contributor to their risk environment. Stigma increases the risk of HIV acquisition to sex workers, yet it is experienced in several forms at the individual, interpersonal and structural levels [14, 16–19].

In Uganda, sex work is illegal and criminalised. Research that investigated the contexts in which women in Kampala sell sex has however shown that women join commercial sex work because of their disadvantaged backgrounds and restricted access to economic resources [11, 20–22]. Mbonye et al. [11] showed that women providing services in outdoor locations like streets, alleys and parking lots faced more challenges than women providing services in indoor locations like nightclubs, bars, and lodges. These challenges included exposure to violence, stigma from the public, and visibility to police [11, 23]. Kawuma et al. [20]reported in a more recent study that the places in which women sell sex in Kampala are fluid in that they move from one type of venue to another. All these studies also showed that women selling sex in Kampala faced violent relationships with both the police/authorities and with their clients [11, 20, 21].

It is however noteworthy that women included in these studies were participants from large epidemiological cohorts that recruited participants from low socio-economic settings, with little or no education, and who typically recruited their clients in physical locations, indoor or outdoor [11, 23]. Women outside of these cohort settings, who have higher education, belong to higher socioeconomic status, and meet clients in spaces other than those identified in these studies have not been included in important HIV research, programming, and prevention efforts in Uganda to date. Research in the United Kingdom, USA, Australia, Japan, and India has reported the experiences of women who sell sex using internet websites and social media platforms [24–26]. These women also face risks, violence, and crime just like their peers who meet clients in physical locations like venues and streets [24, 27]. Understanding the contexts in which women sell sex and the strategies that they use to advertise, meet, and provide services to their clients will help us to understand HIV risk among women by highlighting how structural, interpersonal, and individual factors interact to influence HIV transmission. In Kampala, earlier studies have reported on the contexts in which women recruiting and providing services in physical locations work, but there is still a gap in knowledge about the prevalence of client recruitment using online platforms, how women who recruit this way are organised, and how this strategy affects their risk of HIV acquisition. Understanding these gaps will improve our understanding of the structural determinants framework for HIV prevention among women selling sex in Kampala. This paper presents a more comprehensive understanding of the contexts in which women sell sex in Kampala by including women who have not been included in prior research studies and emphasizes the need to reach them and target intervention efforts to them. This aligns with the UNAIDS strategy of leaving no one behind and reaching the populations at the greatest need of care [28].

## Methods

### Study design, participants, and process

Twenty women from Kampala and surrounding suburbs were included in the study, using two sampling strategies. The first sample – the clinic sample – included 10 women sampled from a cohort of 4500 women who had been attending a clinic dedicated to women at risk of HIV acquisition including FSWs run by the Medical Research Council/ Uganda Virus Research Institute and London School of Hygiene & Tropical Medicine (MRC/UVRI & LSHTM) Uganda Research Unit in Kampala [23]. Women who met clients in physical spaces like venues and outdoor locations had prior been recruited into the clinic through peers. The second sample – the snowball sample – included 10 women who met clients using online platforms including social media and websites such as Instagram. We identified one key informant who started the snowball recruitment as described by Heckathorn [29] and Rao et al. [30]. Women were eligible to participate if they were 18 years or older, self-identified as sex workers or offered sex for money and spoke Luganda or English. In our study, “women who meet clients” includes women actively recruiting clients, women searched out by clients, and women who are introduced to clients by peers, but meet using online spaces.

### Data collection and management

An experienced female graduate social scientist (MH) made first contact with all women, planned interview appointments, administered the study information and consent process, and carried out in-depth interviews with them. For the clinic sample, we selected women from the cohort using a random number generator in Microsoft Excel to generate 10 random numbers within the range of 1 and 4,500 inclusive, which matched the women’s unique cohort identifiers. We invited women with the corresponding numbers to participate. To identify the seed for the snowball sample, the female social scientist (MH) used the Instagram search function to search through posts of women who offered mobile (in-house) massage services or sex for money. She used the keyword “massage” and the location filter set to “Kampala”. The results included both personal accounts and accounts for massage parlors. We considered the first personal account that appeared on the search results as the potential seed for our sample. The female social scientist (MH) contacted the first personal account via the Instagram chat function, providing information about the opportunity to participate in a research study. The owner of the personal account agreed to take part in the study. After her interview, the seed identified through Instagram identified other women and provided their contacts. The female social scientist (MH) then invited the potential participants to the study, and the snowball continued until 10 interviews were completed. We allocated participant numbers from A01 to A10 for those in the snowball sample, and B01 to B10 for those in the clinic sample. Interviews were carried out between September and October 2022.

We developed the interview guide from a literature review of the structural factors that influence HIV prevention for women who sell sex, and a review by Shannon et al. [6], which presented a framework for the structural drivers of HIV and the pathways through which they interact with interpersonal and individual behavioural factors. This framework expanded structural factors to include macro-structural factors such as legal, socio-political, cultural, economic, and geographic contexts in which women sell sex, sex work organisation which includes the organisational structure, community empowerment and collectivisation of sex work, and the work environment which includes the physical, social, economic and political features of the environments in which sex workers operate, such as violence, access to condoms and anti-retroviral therapy (ART), and venue policies [6]. Using this framework, we developed this guide specifically for this study, and included questions on how women joined and why they would leave sex work, how their work was organised including recruitment and where they provided services to clients, their relationships with clients and authorities, the illegality of sex work, and the stigma they experienced. A copy of this interview guide is included as an additional file (see Additional file 1). We collected basic demographics at the beginning of the interview, asking women about their age, number of children, level of education, if sex work was the main occupation, and if they used social media to meet men for sex work. These were summarised in MS Excel, and the corresponding frequencies presented as descriptive statistics. Recruitment logs with personal information were stored in a secure access-controlled cabinet separate from where interview notes, recorders and computers were kept. After obtaining informed consent from the participants, we audio-recorded interviews, then transcribed and translated them into English. The social scientist (MH) took notes to back up the recordings. We imported the transcripts, translations, and interviewer notes into NVivo 12 for data organisation and management.

### Data analysis

We used framework analysis as outlined by Gale et al. [31] to analyse the qualitative data. This analytical approach involves developing a thematic structure for interpretation, under which individual codes can be grouped and compared [31].

A study team member checked five random transcripts in English for transcription accuracy, and all the 10 Luganda transcripts for translation accuracy. In the first step of the coding, both the first author and the social scientist (MH) coded four interviews independently using initial frameworks constructed both deductively using the review by Shannon et al. (2015) and inductively using themes arising from the interviews [3]. The two coders then met and consolidated their coding frameworks into a revised version, which the first author used to finalise coding of all the interviews. From the consolidated coding framework, we developed a framework matrix with the themes and subthemes as the columns, and the participants as the rows. We populated the cells of the matrix with both summaries and representative quotes from the data. We then analysed the data from each of the columns to generate analytical memos on prominent themes arising from the data. All the steps of the analysis were reviewed by two other co-authors.

### Ethical considerations

This study was approved by the Uganda Virus Research Institute Research and Ethics Committee (GC/127/912), the Uganda National Council for Science and Technology (HS2386ES), and the ethics committee of the London School of Hygiene and Tropical Medicine (28,175). We obtained written informed consent from all the respondents before data collection. We compensated the participants 20,000 Uganda shillings (UGX), (USD 5.5) for their time, and 20,000 UGX (USD 5.5) for their transport. We did not offer current participants any incentive to refer seeds and informed them that they would not face any penalties whatsoever if they did not refer any seeds. To contact new participants for the snowball sample, the qualitative researcher was provided with a partial name and a contact number, or with the new participant’s Instagram handle. The identity of the referring participant was not disclosed to new participants. The referring participant was not told which of the potential participants suggested by her eventually participated in the study. A copy of the script we used is included as an additional file (see Additional file 2).

## Results

### Women in our study

Twenty women participated in the study, 10 in each of the clinic and snowball samples. Of the 20 included women, 12 met clients using online platforms. Of these 12, nine were from the snowball sample and three were from the clinic sample. We reached out to 26 women for inclusion in the snowball sample, eight of whom opted not to participate, six did not come for their appointments, and two did not respond. In the clinic sample, only one of the 10 women was not reachable and was replaced. While women from the clinic sample generally had less schooling and were predominantly unmarried, women from the snowball sample generally had high levels of education, had professional jobs or were students in training for professional jobs, were able to negotiate better prices for sex, and were able to avoid outdoor confrontation with police, authorities, and the public. Table 1 below gives details of women’s individual characteristics.

**Table 1:** Characteristics of women in our study

Sample	1 – Clinic (*n* = 10)	2 - Snowball (*n* = 10)
**Age in years**		
23–25	1	3
25–30	6	7
> 30	3	0
**Highest level of education**		
None	1	0
Primary 7	7	0
Senior 4 (UCE)	2	0
Senior 6 (UACE)	0	1
Diploma	0	1
Bachelor’s degree	0	8
**Met clients using online platforms**		
Yes	3	9
No	7	1
**Has children**		
Yes	10	3
No	0	7
**Sex work is her main occupation**		
Yes	8	4
No	2	6
**Has a regular partner**		
Yes	7	5
No	3	5
**Interview language**		
English	0	10
Luganda	10	0

### The contexts in which women sold sex

The prominent themes we identified in our study included: how the women organised their work, why and how they joined or would leave sex work, the relationships that they had with clients, authorities, family, and their peers, and the stigma they experienced. We present them in Table 2 below and explain them in detail in the sections that follow.

**Table 2:** The contexts in which women sold sex in Kampala

Theme	Sub-themes	Evidence of sub-themes
Entry into and exit from sex work	Reasons women joined sex work	Economic need, experience of sexual violence
	Reasons women remained in sex work	Economic responsibility towards dependants (e.g., children)
	Reasons why women would leave sex work	Stable comparable income, Marriage
How female sex work was organised	Where women met their clients	Private virtual online spaces, public spaces, ‘pimps’
	Where women provided services to clients	Private indoor (homes), public indoor, public outdoor
	Competition in service	Where they met clients, individual physical attributes, information held about clients
	Negotiations with clients	Online vs. in person negotiations
Nature of relationships women had	With authorities	Violence, abuse, and exploitation, protection in some cases
	With clients	Violence, abuse, support, and friendship in some cases
	With peers	Jealousy, mistrust, hatred, friendship in some cases
Women’s experiences of stigma	None	Internalised, perceived, and discrimination

#### Reasons women joined sex work, and why they would leave

Women mentioned economic need as the main reason for joining sex work, and this was driven by the loss of parents, abandonment by partners, economic hardships due to the COVID-19 pandemic, inability to continue school due to lack of school fees, and costs like rent and food.

*I joined sex work because of the hardship I was going through after my husband abandoned me and the children, he was not paying their school dues, and they had nothing to eat. So, I decided to devise means of survival.* (Clinic sample, 23–25 years, B04).

Women remained in sex work because of economic responsibilities and no alternative sources of comparable income. For women who met clients in public spaces, these responsibilities included costs such as rent, school fees and food for themselves and their families. For women who met clients using online spaces, responsibilities included special costs such as maintaining their lifestyle and good aesthetics both on online platforms and the social scene. They included rent for expensive apartments, hairstyles, makeup, expensive clothing and phones, trips outside Kampala and Uganda, and keeping up appearances on the Kampala party scene.

*At this point as much as the money you get from sex work is little if I decide to leave, I won’t be able to sustain myself or even be able to start another business since I will not have money. The situation is bad these days, so if I leave sex work, which other job am I going to do?* (Clinic sample, 23–25 years, B04).

*The money that it comes with is not little money. This is like salaries that people get for months, and I am doing it for just one day. So, it becomes addictive, and you must keep up with the lifestyle that you have started so you must keep going back until you are somewhere that you want to be.* (Snowball sample, 25–30 years, A04).

While all participants mentioned economic need as reason for joining or staying in sex work, some women joined sex work because of trauma from being abused as children. The pain that they harboured from this trauma kept them in sex work, even if they were not proud of their work. Regardless of how they joined sex work or where they met their clients, most women would leave sex work if they had major changes in their social or financial status, for example if they got married, achieved financial stability through stable alternative and comparable sources of income, or having a home that they own.

*Oh well yeah one day I want to have a family settle down and have a husband and have kids so definitely there is no way I can be married to someone when I am still doing this kind of work.* (Snowball sample, 25–30 years, A04).

#### How female sex work in Kampala was organised

##### Where women met clients and provided services

Women discussed recruiting clients in public physical spaces, in private virtual online spaces, and through go-betweens. The public spaces were both outdoor and indoor. Outdoor public spaces included streets, alleys, and markets, while indoor public spaces included venues such as bars, pubs, cafés, offices, churches, malls, casinos, hotels, restaurants, massage parlours and lodges. Women also discussed the lack of privacy and the higher risks of police prosecution and arrests, attacks by thugs, robbery, and exposure to judgement by the society, in addition to meteorological challenges like cold, windy, and rainy weather.

*The person who took me on the streets [a female friend], one time we were on the street and her [the friend’s] uncle was the one haggling with her. (Laughs). Those are the things that make us leave the streets. At least you go to [the clients’] places or at our [the woman’s] place it has no problem.* (Clinic sample, > 30 years, B02).

Women discussed benefiting from security offered by the management of indoor public spaces, even if in some cases they were charged a fee to be allowed to work at these places.

*The street is not good but at the bar they first check clients before entering, they do not allow them to enter with keys, knives and other things which is not done on streets. That is why you see that many people who work from streets die a lot, that is why the street near [a pub nearby] many people die from there…For the places, I told you like [a specific pub], it is safe, even if a client becomes chaotic, we are protected by the guards at the bars.* (Clinic sample, > 30 years, B03.

The private virtual spaces mentioned by women were online platforms that can be accessed from their homes, or other private and protected places. They included social media applications (apps) and sites such as Snapchat, Instagram, Badoo, and dating websites. Women who met clients using these spaces were able to reach many clients, had more time between the first contact with a client and accepting to offer services to the client. This time allowed them to make decisions both about their perceived safety with clients and avoid potential violent clients, but also about HIV prevention. They earned more than their peers who met clients in public spaces, and they provided services mostly in hotels, in the clients’ homes, and sometimes in their homes.

*The advantage of hotels is that you can easily get help in case of any problems, which you can’t get when you are in someone’s home because its already night and some people’s homes are fenced even if you shout no one can help.* (Snowball sample, 25–30 years, A07).

*Well, the truth is there is a lot going on, on social media. When you get offers, it is up to you to take them or not. Social media things are so easy now. You can meet people; you can easily associate with people from different parts of the world.* (Snowball sample, 25–30 years, A02).

Women who met clients in virtual spaces faced some challenges particular to their strategy of recruiting clients, for example cyber threats and their online accounts being hacked into, new clients who did not want to pay being extorting money from them, and old clients who traded women’s confidentiality for money.

*Because I had so many people writing to me. They wanted to meet me. So, I felt like Instagram wasn’t a safe place for me. And by then people used to hack into accounts.* (Snowball sample, 25–30 years, A01).

Some women relied on *pimps* or peers who acted as go-betweens procuring clients for them. These women were assured of a reliable flow of clients from middle and high socio-economic status; and of more security since the go-between knew which woman was with which client, and at what location. However, they were prone to exploitation since the go-between usually took a commission off the women’s pay, while some protected violent clients.

*Well, first there are what they call pimps who usually have contacts of men. Some are like delegates who come to Uganda, or who want to take girls outside for meetings outside of Uganda. These pimps are always looking for sex workers you don’t even have to look for them.* (Snowball sample, 25–30 years, A04).

As much as some women used only private online spaces, others had a primary space where they usually met clients, and one ‘filler’ space they would resort to in case they didn’t have enough clients from their primary space. For example, women from the clinic sample mostly relied on online spaces during day, but used go-between or went out to clubs and bars in the night. On the other hand, women from the clinic sample relied heavily on physical spaces to recruit their clients.

*During the day you can be on your phone, but you must go to clubs at night. If you are in another country, you can’t just stay in the house and chat on phone, you must go outside and look for clients if you need money.* (Snowball sample, 25–30 years, A07).

Women who met clients using online platforms provided services in indoor spaces like their own and clients’ homes, and in hotels, but never mentioned offering services in public outdoor spaces. On the other hand, women who met clients in public outdoor spaces like streets provided services in indoor spaces, but also in the outdoor spaces where they met the clients.

##### How women competed for clients

Women who met their clients in public spaces viewed their counterparts who met clients using online platforms to be in a higher income and of a higher socio-economic status. The latter women discussed that the former operated a more versatile, more mobile, and less exposing form of sex work which was able to attract a clientele of higher socio-economic status and higher paying. Among women who met clients in physical spaces, women who met clients using online platforms were referred to as *bikapu* (plural for *kikapu*) sex workers. A *kikapu* is a large travel or shopping basket that can be carried anywhere at any time, and whose contents are known only to the owner.

*There are sex workers whom you will never see seated in corridors waiting for clients or even see clients entering her house. But she is also at her home doing sex work. If a client calls her, she goes, services the client, and returns to her house. They are always called ‘bikapu’ sex workers.* (Clinic sample, 25–30 years, B05).

##### The prices women charged, and how they negotiated with clients

It was clear from the interviews that women who met clients using online spaces charged more than women who met clients in public spaces. Among women who met clients in public spaces, the highest amount received for a sexual act was 100,000 UGX (USD 27), compared to 40,000,000 UGX (USD 10,767) for those who met clients using online spaces. The latter had a minimum reserve price of 250,000 UGX (USD 67), compared to no payment or providing sex on credit among the former. Moreover, those recruiting online had more time to negotiate prices and compare offers from clients before meeting clients physically, compared to the former, who usually negotiated with one client at a time and when they had already met physically.

*I can even get 8 million shillings. The lowest I get in a month is 5,000,000 shillings [USD 1,356] but it’s usually between 8 and 15 million shillings [USD 2,170–4,069]. When people who live abroad are around in large numbers, I can get up to 15,000,000 UGX [USD 4,069].* (Snowball sample, 25–30 years, A06).

*There are those sex workers who cannot come to my place where I work, but they meet their clients using the internet and somehow charge more expensively than me. I cannot compete with them; I am cheaper because I charge from 5,000 UGX [USD 1.40] but those sex workers charge from 100,000 [USD 28] or 200,000 UGX [USD 54].* (clinic sample, > 30 years, B03).

*You can get a customer who runs away after getting the service as agreed. That is what they call ‘bidding farewell with a zip’ (okusibuza zip). It depends, there is when we work tirelessly and you get 30,000–50,000 shillings [USD 8.20–13.60] monthly, and between two to three thousand (54–81 cents) daily.* (Clinic sample, 25–30 years, B01).

Moreover, women who met clients using online spaces discussed being offered substantial non-financial incentives in addition to cash payment. In most cases, these incentives, which included gifts and trips within and outside Uganda, supplemented the cash payment clients offered and influenced women’s decision to reconsider some clients that had been rejected because the initial payment offer was deemed unattractive.

#### The relationships women had with authorities, clients, and peers

Women faced violence from clients in form of physical, verbal, and sexual abuse such as rape, clients removing or tearing condoms intentionally, and even death threats.

*For me a man almost killed me. We went into a room, and I told him the amount of money I wanted. He said he did not have it. I told him to let me get out, but he started strangling me. Then I accepted that he had robbed me.* (Clinic sample, > 30 years, B02).

*Ah God (covers her face with her palms and shakes her head) it was so hard for me. He slapped me, did everything you can think of. My dear, I gave up and had to act soft because some clients need you to be submissive. So, you must act like you are enjoying whatever he wanted.* (Snowball sample, 25–30 years, A06).

However, some women met friendly and supportive clients who treated them well, got them business connections and supported them financially in their personal lives.

*I will not lie to you; he was taking care of me just like any other man takes care of what he loves.* (Snowball sample, 25–30 years, A01).

Women’s relationships with peers were usually characterised by jealousy, mistrust, hatred, and threats. They fought with each other verbally, physically, and spiritually with witchcraft. That said, there was evidence of friendships among women who met clients in physical spaces. For example, they could demand their peers’ release if they witnessed their arrest.

*First, a massage parlour has a lot of girls. So, there is that hatred that comes along. Then there is a risk of being bewitched by those girls at the parlour.* (Snowball sample, 23–25 years, A08).

*Yes, there are sex workers who compete against each other. I don’t know how to explain this but sometimes your fellow sex workers might notice that you are getting a lot of customers then they go and bewitch you.* (Clinic sample, 23–25 years, B04).

Women who met using online spaces worked in isolation and were in many cases not able to get help in cases where clients turned violent. And because sex work is illegal in Uganda, women had no legal support or protection from authorities. Instead, they were exploited sexually and financially by the authorities, abused, and violated. All our participants faced some form of violence, abuse and exploitation from police and authorities.

*We are treated badly. Police officers also come and arrest you and sometimes even rape you. Sometimes when they arrest you and you don’t have money to give, they force you to have sex.* (Clinic sample, 23–25 years, B04.

*They all want sex (laughs). The truth is I don’t want to say everybody is bad among authorities but it’s like they all want to get something [sex]. Of course, I don’t give them, but I am sure there are people who do.* (Snowball sample, 25–30 years, A02).

Authorities only offered protection when they got sexual favours from women, and when women paid regular fees to them. Women working in private indoor spaces like pubs discussed being protected from clients that turned violent, by private guards stationed at these indoor spaces.

Women who met clients in physical spaces were more affected by the illegality of sex work compared to their peers who met clients using online platforms. The former discussed restrictions on the areas or times when they could work, being exposed to arrest by authorities, and public shame and ridicule. The latter women discussed not knowing any laws against sex work, and their work not being hindered in by any regulations. However, majority of the women discussed not being able to report to authorities or disclose to friends and family in cases where they had been raped, for fear of prosecution, ridicule, and stigmatisation.

#### Women’s experiences of stigma

Our participants experienced internalised stigma where they felt like disappointments to their families, and unworthy of some things or levels of achievement in life, such as good loving relationships respect, and leadership positions in society. Some women thought they would only be able to fit in society if they left sex work. Otherwise, they had to live with persistent guilt, shame, and embarrassment from doing sex work, and consequently keeping their work secret from friends, family, and society.

*Then there is also that persistent guilt of letting down your family and them expecting better. I don’t know but it’s embarrassing, how do you even start telling someone that you are getting money from having sex with multiple people not even one.* (Snowball sample, 25–30 years, A07).

Women experienced stigma when they were shunned by their family and friends, health workers, local leaders, and the communities in which they live and work. They were pushed to operate in secrecy because they feared the stigma they would face if exposed. Women who met clients in public outdoor spaces like streets were most affected because they were more exposed to the public while working, and to arrests by authorities.

***Banvuma****[They insulted me]. I remember my mum told me I decided to go out and embarrass the family, yet they have degrees and masters. It was really bad. I never got invited to any family function. Ever since then I became a reject, and you know you can tell when you are rejected by how people look at and talk to you.* (Snowball sample, 25–30 years, A01).

*Yes, from the neighbours one of them can see you or in a way find out that you do sex work. Then she comes and tells another person that you are a sex worker. Then they spend the whole day gossiping about you.* (Clinic sample, 25–30 years, B05).

Women discussed not being able to get licences since their work is illegal, and not being able to report in cases where clients violated them. They were exposed to discrimination because they had no legal or structural backing for them to work or to be protected against violence, attacks, and exploitation.

## Discussion

We present the contexts in which women selling sex in Kampala met and provided services to their clients. Our participants met clients in physical spaces including venues and outdoor locations and using online spaces that included social media applications and websites. Earlier studies also found that women who sell sex in Kampala recruit clients in venues and outdoor locations like those we presented [11, 17]. Our study goes a step further and highlights that some women met clients using virtual online spaces like social media platforms and websites. While this finding is new to literature on Uganda, it is consistent with studies carried out in other settings, where sex workers recruiting clients using online platforms like social media and websites were identified [24, 25, 27]. similarly to their peers who recruit clients from physical spaces, women who recruit clients using online platforms are also high-risk population, yet they have not been targeted in HIV prevention efforts. There is need for inclusion of women who recruit clients using online platforms in HIV prevention interventions.

We assert that women selling sex in Kampala work in settings where sex work is illegal and criminalised, and because of this they are forced to endure harsh treatment; they face violent and abusive clients; they are arrested, abused, and exploited by authorities; and they experience jealousy and violence from their peers, and stigma from society. It is known that sex work is illegal in Uganda, that women who sell sex have violent relationships with both clients and authorities, and that women selling sex get no legal protection [17, 21]. Our findings are consistent with other studies in this respect. We go further and highlight the larger extent to which the illegality of sex work was felt by women who met clients in physical spaces compared to those who met clients using online platforms. This stresses the continued need for support to women who face violent relationships, and to create safe spaces for women selling sex.

We also show that women who met clients using online platforms had more time to engage and negotiate with the clients before meeting them physically, were able to generate a pool of potential clients and consequently had less pressure to find clients. These women also seemed to have better education and income compared to their peers who met clients in physical spaces. Despite these apparent individual level advantages, we show that in many ways women selling sex faced similar pressures at the structural and interpersonal levels and faced similar risks with regards to HIV acquisition.

All our participants faced challenges that are similar and consistent with those identified in earlier studies [11, 17, 20, 21, 23]. These challenges were sustained by gaps in structural, social, and interpersonal support with regards to HIV prevention. For example, all study participants were either unable or unwilling to obtain support from authorities in situations where they were abused, exploited, or violated by clients or authorities. Women who met clients using online platforms faced some challenges specific to them because of their client recruitment strategy. First, they had to deal with cybersecurity threats like their social media accounts being hacked into and being exposed on the online platforms where they met clients. The damage caused by such negative exposure would be amplified by information on these platforms being easily and affordably accessible to very many people simultaneously. Secondly, they were threatened with exposure and reputational harm by clients who did not want to pay for services. This further increased their already high costs of operation. In terms of risk, most women who met clients using online platforms were unable to get immediate help in case a client turned violent because they mostly provided services to clients in their homes (both the clients’ and women’s) and in hotels. These women were exposed to high levels of violence that was potentially fatal from clients, and yet they did not readily access the needed services because they were pushed to operate in secrecy due to fear of stigma, judgement, and prosecution. This was exacerbated by the fact that they were mostly university graduates with professional jobs and were therefore very secretive and protective of their involvement in selling sex. Women’s experiences of stigma were consistent with what has been found in the literature (Beattie et al., 2023; Cruz, 2015; Fitzgerald-Husek et al., 2017; Ruegsegger et al., 2021; Seeley et al., 2012). It is still interesting to note that our participants across the samples faced stigma in similar ways and that most were ashamed of their work. Even women who met clients using online platforms were unable to report clients because they feared the prosecution by authorities or judgement by society that would come with being exposed. Provision of safe structural and social environments that support and protect women who sell sex as they carry out their work is necessary. Additionally, interventions to reduce stigma for women who sell sex are still very important but should target the more secretive and protective women who recruit clients using online platforms.

While access to health care for women who sell sex has improved over the years, these improvements in access have been identified among women who sell sex and have been included in research studies. This includes women in the clinic sample of our study, who mostly meet clients in physical spaces. Access to health care and HIV prevention services for women who meet clients using online platforms has not been systematically recorded. Yet, our results show that women who meet clients using online platforms face similar and even more challenges than their peers who meet clients in physical spaces. While the common challenges that all women face, including stigma and violence are barriers to health care access [32–34], the additional challenges that women who meet clients using online platforms face could be additional barriers for access to health care. This calls for continued efforts to address the common challenges but also highlights the need for specific interventions to improve access to health care among women who meet clients using online platforms. Our findings on how women joined sex work or would leave are consistent with published literature. Earlier research showed that women joined due to economic need, or because of earlier traumatic experiences of sexual abuse, and they would leave if they achieved economic stability [16, 21, 35]. This further highlights the importance of continued efforts to empower all women, and protect them from sexual violence, regardless of their level of education, status of work, and where they recruit or provide services to their clients.

Women who met clients using online platforms were hard to reach for us as a research team, and we assume that it will be hard for other researchers, health service providers and policy to reach them effectively. In fact, most women who we contacted to be part of the snowball sample (16 of 26) did not participate in the study, and those who accepted did so with caution. The spaces in which our participants provided services were identical to those reported in the literature, i.e., in indoor venues and outdoor locations [11, 17, 20]. We however highlight the fact that women who met clients using online spaces always provided services in indoor spaces and never in public outdoor spaces. Intervention efforts that target women recruiting clients in venues and in outdoor spaces will therefore miss women who recruit using online platforms. To increase their access to health care, to support services, and to the HIV prevention services they need, research and policy makers need to generate innovative strategies that will reach and engage women recruiting clients using online platforms.

### Strengths and limitations

We used the framework analysis method. This method can neither handle highly heterogeneous data nor pay attention to the language of the respondents and how it is used [31]. We could therefore have missed some heterogeneity in women’s individual, interpersonal, or structural factors because of our choice of data analysis method. Moreover, we based our initial interview guide and coding framework on structural factors identified in the literature. Even though we used some inductive coding to complement the initial deductive framework, results from a similar study using a fully inductive approach would make an interesting comparison. We neither used complex theories nor sought to develop theory derived from the data but used robust framework analysis techniques to generate the major themes related to the structural factors that affect the sexual and reproductive health of women selling sex in Uganda. Despite these limitations, we present important results that could be applicable to women selling sex in Uganda, and other similar settings.

## Conclusion

Over half of women in our study met their clients using online platforms and faced additional specific challenges and risks by recruiting their clients using online platforms. Regardless of where they met their clients, our participants worked in environments that exposed them to high risk of acquiring HIV. To reduce risk of HIV acquisition among women who sell sex, researchers and implementers should consider these differences in contexts, challenges, and risks, and design innovative interventions and programs that reach and include all women selling sex in Kampala.

### Electronic supplementary material

Below is the link to the electronic supplementary material.

Supplementary Material 1

Supplementary Material 2

## Data Availability

The datasets used and/or analysed during the current study are available from the corresponding author on reasonable request.

## Abbreviations

FSW: Female Sex Worker
LMIC: Low and Middle Income Country
HIV: Human Immunodeficiency Virus
ART: Anti-Retroviral Therapy
UGX: Uganda Shillings
USD: United States Dollars
COVID-19: COrona VIrus Disease of 2019
MS: Microsoft
