# Supplementary material for: Understanding the contexts in which female sex workers sell sex in Kampala, Uganda: a qualitative study
Source: BMC Womens Health. 2024 Jun 26;24:371. doi: 10.1186/s12905-024-03216-7 (PMC11202390; doi:10.1186/s12905-024-03216-7)
Supplement: Supplementary file 1 — Supplementary Material 1 [file 12905_2024_3216_MOESM1_ESM.docx]

Hello good morning, my name is ‘*Qualitative Researcher’*, a social science research assistant working with Medical Research Council Entebbe.

We are carrying out a study about aspects that affect women’s health in Kampala, and we would like to carry out interviews with some women who have the relevant experiences and information about the specific subject of the study.

Your contact was given to us by someone who was already interviewed, and I would like to request for your availability so that I can tell you more about the study, what it entails and then you can decide for yourself if you would like to be part of it or not.

(Check for phone or physical availability, **but physical availability first**)

We have offices in Kampala at the Aids Information Centre, and in Entebbe at Uganda virus research institute, but can meet up in a place that is convenient for you, so you can also suggest any other place of your preference if these are not ideal for you.

You will be facilitated for your time and transport (*for the interview only*). So, I suggest you remove this because someone will come and say they won’t participate, and then you still have to refund!
