# Supplementary material for: Understanding the contexts in which female sex workers sell sex in Kampala, Uganda: a qualitative study
Source: BMC Womens Health. 2024 Jun 26;24:371. doi: 10.1186/s12905-024-03216-7 (PMC11202390; doi:10.1186/s12905-024-03216-7)
Supplement: Supplementary file 2 — Supplementary Material 2 [file 12905_2024_3216_MOESM2_ESM.docx]

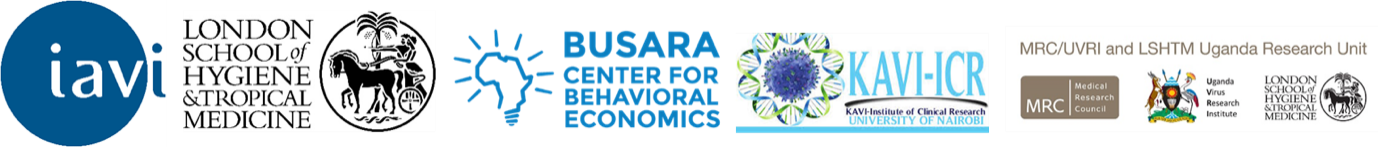

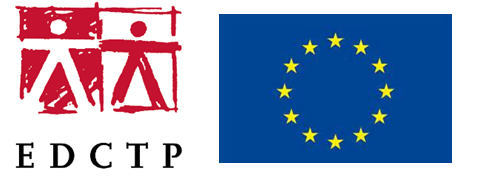

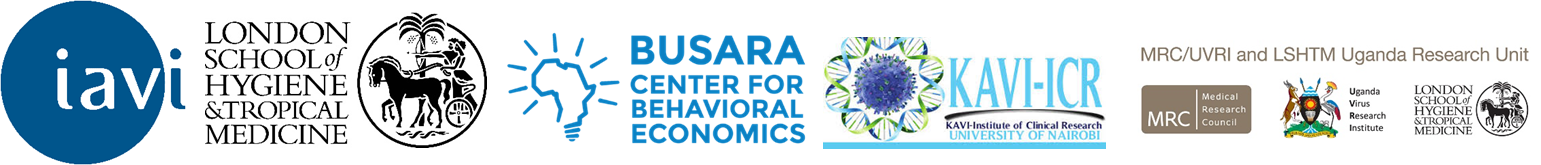


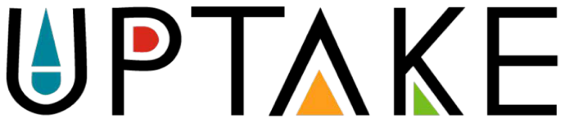


**The Role of Structural Factors and Behavioural Traits in HIV Prevention among Female Sex Workers in Kampala, Uganda: A sub study of the UPTAKE study**

| **Interviewer** |  | **Date** |  |
| --- | --- | --- | --- |
| **Venue** |  |  |  |

**INSTRUCTIONS FOR THE MODERATOR – How to use this IDI Guide**

1. This guide has 3 levels of questions:

- Numerical research questions/topic areas highlighted in grey. These are the research questions/areas that we want to get answers to. They are guides that don’t need to be read out aloud.
- Discussion questions: the questions that you as the Interviewer **will ask** respondents in order to get answers to the research questions. **These questions will be underlined and in bold.**
- Probes: the bullet points below the discussion questions. The interviewer should ensure that key topics listed in the probes have been addressed/discussed during the interview. So, depending on what has already been discussed you may ask these probes or not.

1. *Instructions/suggestions to interviewer are in italics and brackets [ ].*
2. The discussion guide is divided into three columns.

- **The left-hand column** contains the research questions, discussion questions and probes. It is not required to read the discussion questions verbatim, but they are written to ensure some consistency across discussion. You may adapt the question, depending on how the discussion develops, but ensure that all the questions have been asked by the end of the interview.
- **The right-hand column** is for summarising the themes brought up by the women in the discussions. These should be summaries of the general issues raised in connection with the research question. These summaries should be more than just yes/no, but not longer than a few sentences of bullet points. They do not need to be detailed, as we have the details on the tape.

**INTRODUCTION**

*[Welcome the participant and thank her for coming. Hand the participant a sociodemographic information sheet.]*

Explain the general purpose of the discussion:

***“The purpose of this interview is to understand the challenges that sex workers face, and how they make choices related to their sex work.”***

*[Address the issue of confidentiality: Inform the respondent that any information discussed will be analysed as a whole and that their names will not be used in any analysis of the discussion.]*

*[Introduce yourself to the participant.]*

*[Have the respondent introduce themselves. They should create a nickname for the discussion. Have them write it down on a sheet of paper that they will keep on their laps and refer to throughout the discussion.]*

*[Ask for any questions.]*

*[Inform the participant that you are going to start recording and reconfirm that they are ok with it.]*

*[Ask “ice-breaker”: Ask the respondent to introduce themselves by their nickname and ask them to share the* ***happiest day of their life and what made them happy about that day.***

|  | **Today we are going to talk about your sex work. None of your personal individual information will be identifiable or disseminated, but rather aggregated findings with no personal data identifiable will be disseminated.**  *[Look out for visual cues at this point and make sure the respondent is convinced of the confidentiality and comfortable before proceeding with the interview. If the respondent is not comfortable, continue to reassure until they are comfortable.]*  **What do you understand when someone says ‘*sex work’*?** |
| --- | --- |

|  | **Research Question, IDI Questions and Probes** | **Summary/Notes** |
| --- | --- | --- |
| **1.** | How do Female Sex Workers join sex work, how do they get clients, and how do they provide the sex to the clients?   - 1. **Can you tell me why you started sex work** - What other options did you have at the time you joined SW? - Why are you working now? - Do you plan on leaving sex work? If no, why not? - If yes, what would make you leave? - Would it be easy to leave? - What advice would you give someone who is looking to join or leave sex work?   1. **Tell me about how you get and serve clients**      - Different sex workers operate in different places: on the streets, in doors, high end escorts. - Where do you pick your clients? - Where do you service your clients? - What do you think are the challenges and opportunities for operating in these different areas? - Some sex workers use social media like WhatsApp, Telegram etc to meet clients. - Do you use social media? - Which social media platform do you use? - Which social media platforms do you use to get clients? - How do you use this platform to get clients? - What do you think are the benefits and challenges of social media?   [*Also probe if people can move within areas and modes of operation to another*]   - 1. **Tell me about other sex workers or people who you think you compete or collaborate with in providing sex work?**     What relationships does the sex worker have with clients, with authorities, society and with other sex workers? |  |

| **2.** | **2.1 Tell me about any form of violence you have experienced in your line of work?**   - Tell me about a violent encounter with a client, authorities or fellow sex workers that you have had, and how this encounter affected your work. - Now, I would like to hear from you what you think the best solution to a violent encounter would be.   **2.2 Tell me about any form of stigma you have experienced in your line of work?**   - Tell me about perceived stigma from family and from society. - Have you faced any stigma from **authorities and in the environment** in which you work? - Have you ever been denied services or care such as health care or social services? - Are there things or services you think you do not deserve from the **society,** **authorities, and environment** in which you work, for example health care or social services? - Now, I would like to hear from you what you think the best solution to the stigma would be. |  |
| --- | --- | --- |
| **3** | How does regulation affect the female sex worker?  **3.1 Tell me about any regulations affecting Sex work that you know of**   - Some places have regulations that can affect sex work. How do regulations in your area affect your work?   **3.2 Please tell me about what you feel you have had to or still have to endure just because sex work is illegal in Uganda**   - Tell me about any pressures that you feel you have to endure - Tell me about any type of clients and situations you are ‘forced’ to pick - Tell me about what sexual activities and behaviour you are pushed to endure |  |
| **4.** | What is the sex worker’s current sexual risk behaviour?  **Please tell me if and how you protect yourself from infections when relating with your clients**   - Tell me about the kind of partners you have - Some sex workers see mostly regular clients, while other see mostly random clients. Which option do you think is better and why? - I would like to hear about any HIV/STI/Pregnancy prevention methods that you are currently using, and those that you have used in the past - Tell me how often you use these prevention methods - If you were to design a new product, what benefits would you give it? |  |
| **5.** | Does the sex worker use any substances during their work?  **Tell me about your experience using any substance before or during sex work**   - Some people use substances before or during their work. Tell me about your experience accessing and using any substances   *[Substances such as drugs and alcohol]* |  |
| **6.** | Does the sex worker face other structural and organisational factors in their sex work?  **What else that we have not talked about would you say about the environment in which you work and live as a female sex worker?**   - Any other information about how you joined or what would affect your exit from sex work? - Any other information about what affects how you identify, pick and service clients? - Any other information about relationships with clients, authorities? - Any other information about stigma from friends and family? - Any other information about regulation both council and national? - Any other structural and organisational challenges that you face in your operation? - Anything or things structural and organisational that make your work and life as a sex worker easier? |  |

**I am now going to ask you some very short and brief questions about your background. These should be short and direct.**

| **Background/ Individual Factors**   1. Highest education level achieved   a None  b PLE  c UCE  d UACE  e Tertiary  f University   1. Marital status ____   a Single  b Married  c Widow/widower  d Cohabiting   1. Number of children ________ 2. Age ____ 3. Is SW your main occupation? ____   a Yes  b No   1. What is your estimated monthly income from Sex work?   _____________ UGX   1. If you answered **“no”** in Q10 above, what is your main occupation?   _______________   1. What is your estimated monthly income from your main occupation in Q10 above?   _____________ UGX   1. How many adults are in your household? _____ 2. What is the estimated monthly income of your entire household? _________ UGX 3. Do you have a regular partner?   a Yes  b No   1. The last time you had sex, was it with a regular partner, or with a client? ______________   a Regular partner  b Client   1. Was your last sexual act protected? ____   a Yes  b No   1. What protection did you use?   _________________   1. If yes, who suggested to use protection? ____   a Myself  b The client  c Both of us   1. If no, who refused the protection?   a Myself  b The client  c Both of us   1. What price do you normally charge for sex with a condom? __________ UGX 2. What price do you normally charge for sex without a condom? __________ UGX |  |
| --- | --- |
